# In-hospital bioimpedance-derived total body water predicts short-term cardiovascular mortality and re-hospitalizations in acute decompensated heart failure patients

**DOI:** 10.1007/s00392-024-02571-7

**Published:** 2024-11-04

**Authors:** Alessandro Faragli, Alexander Herrmann, Mina Cvetkovic, Simone Perna, Eman Khorsheed, Francesco Paolo Lo Muzio, Edoardo La Porta, Lorenzo Fassina, Anna-Marie Günther, Jens Oetvoes, Hans-Dirk Düngen, Alessio Alogna

**Affiliations:** 1 Department of Cardiology, Angiology and Intensive Care Medicine, Deutsches Herzzentrum Der Charité, Campus Virchow-Klinikum, Augustenburgerplatz 1, 13353 Berlin, Germany; 2 Berlin Institute of Health (BIH), Berlin, Germany; 3 DZHK (German Centre for Cardiovascular Research), Partner Site Berlin, Germany; 4 Department of Cardiovascular Surgery, UKE- Unversitätsklinik Hamburg Eppendorf, Hamburg, Germany; 5 Division of Human Nutrition, Department of Food, Environmental and Nutritional Sciences (DeFENS), Università Degli Studi Di Milano, Milano, Italy; 6 Department of Mathematics, College of Science, University of Bahrain, P.O.Box 32038, Sakhir, Kingdom of Bahrain; 7 Division of Nephrology, Dialysis and Transplantation, Scientific Institute for Research and Health Care, IRCCS Istituto Giannina Gaslini, Genoa, Italy; 8 Department of Electrical, Computer and Biomedical Engineering, University of Pavia, Pavia, Italy; 9 Department of Bioengineering, University of California, Los Angeles, USA

**Keywords:** Heart failure, Congestion, Bioimpedance analysis, Machine learning, Cardiovascular mortality

## Abstract

**Background:**

Hospital re-admissions in heart failure (HF) patients are *mostly* caused by an acute exacerbation of their chronic congestion. Bioimpedance analysis (BIA) has emerged as a promising non-invasive method to assess the volume status in HF. However, its correlation with clinically assessed volume status and its prognostic value in the acute intra-hospital setting remains uncertain.

**Methods and results:**

In this single-center observational study, patients (*n* = 49) admitted to the cardiology ward for acute decompensated HF (ADHF) underwent a daily BIA-derived volume status assessment. Median hospital stay was 7 (4–10) days. Twenty patients (40%) reached the composite endpoint of cardiovascular mortality or re-hospitalization for HF over 6 months. Patients at discharge displayed improved NYHA class, lower body weight, plasma and blood volume, as well as lower NT-proBNP levels compared to the admission. Compared to patients with total body water (TBW) less than or equal to that predicted by body weight, those with higher relative TBW levels had elevated NT-proBNP and E/e´ (both *p* < 0.05) at discharge. In the Cox multivariate regression analysis, the BIA-derived delta TBW between admission and discharge showed a 23% risk reduction for each unit increase (HR = 0.776; CI 0.67–0.89; *p* = 0.0006). In line with this finding, TBW at admission had the highest prediction importance of the combined endpoint for a subgroup of high-risk HF patients (*n* = 35) in a neural network analysis.

**Conclusion:**

In ADHF patients, BIA-derived TBW is associated with the increased risk of HF hospitalization or cardiovascular death over 6 months. The role of BIA for prognostic stratification merits further investigation.

## Introduction

Already termed as an epidemic in 1997, 23 million people were estimated to suffer from heart failure (HF) in 2010 [1]. By 2017 [1], this number has almost tripled and is expected to grow at a rapid pace due to population aging [1], with projections showing that HF prevalence will result in more than 80 million diagnosed patients in 2030 [2]. The large number of patients suffering from HF and especially the high number of re-hospitalizations, aggravates the current scenario [3]. Indeed, as much as 47% of HF patients suffer from at least one re-hospitalization within the first 6 months after an initial hospitalization for acute decompensated HF (ADHF) [3, 4]. Of those patients, 90% of cases are caused by an acute exacerbation of chronic fluids congestion [5, 6], a deadly complication that leads to pulmonary edema and, if left untreated, to death [5, 6]. This cascade of repeated re-hospitalizations significantly reduces the quality of life in HF patients, thus, earlier detection of potential congestion for the prevention of decompensation events has never been more urgent [7].

Different invasive and non-invasive solutions have been developed and tested aiming to predict and prevent decompensation events in HF patients [8, 9]. One of the most effective solutions has been the CardioMEMS pulmonary artery pressure monitoring, which has recently shown to improve the quality of life and reduce heart failure hospitalizations in patients with chronic moderate-to-severe HF [10]. However, its adoption has been limited due to the invasiveness and lack of data on its cost-effectiveness [11, 12]. Several non-invasive solutions are available for monitoring parameters such as blood pressure, blood glucose, oxygen saturation, and ECG [13], while only a few are currently available on the market for the assessment and early detection of congestion status in homecare settings. Patients are generally advised to assess their weight with standard weight scales, and changes in body weight (BW) are interpreted as a surrogate estimation index of changes in the volume status [5]. Despite a poor diagnostic and prognostic accuracy [14, 15], the monitoring of BW is still a widely adopted method to guide diuretic therapy and clinical decisions, not only for home-care but also during acute hospitalizations [14, 16].

Bioimpedance analysis (BIA) has emerged in the past decades as an accurate, safe and non-invasive method to assess the volume status of patients [17, 18], especially during dialysis in end-stage chronic kidney disease [19, 20], and in the intensive care setting [21]. In HF patients, BIA has been shown to be useful for diagnostic purposes and has been tested in few prospective studies [22–25]. Nonetheless, its correlation with clinically assessed volume status and its prognostic value in the acute intra-hospital setting are still not established [26]. In the current prospective, observational study, we hypothesized that BIA-derived total body water (TBW) predicts the risk of adverse outcomes in ADHF.

## Methods

### Study population

Patients admitted to the cardiology ward (Charitè University in Berlin—Campus Virchow Klinikum) for a primary diagnosis of ADHF were enrolled. The study conforms to the principles outlined in the Declaration of Helsinki, all locally appointed ethics committees approved the research protocol (EA2/220/19), and written informed consent was obtained from all patients. Demographic, clinical, and laboratory data were recorded within the clinical routine. During hospitalization, a daily assessment of the clinical status, vital parameters, echocardiographic parameters, BW and bioimpedance analysis (BIA) was performed. To minimize slight variations due to respiration, at least 3 impedance measurements were taken per visit and the result was averaged. Peripheral edema was defined by an anamnestic and qualitative increase in leg circumference with a test for a “pitting” edema. Only edema with persisting indentation was considered peripheral edema. Patients with central accumulation of fluid including both pulmonary congestion and pleural effusion determined at admission X-ray. Nt-proBNP was assessed at admission and before hospital discharge. Individual patient treatment followed recommendations of the latest ESC heart failure guidelines [27] and was at the discretion of the treating physician. There were no restrictions on diagnostic or therapeutic options for participating patients. Patients were followed up at 6 months after hospital discharge with a dedicated ambulatory visit or, when not possible, with a telephonic follow-up. The visit included a thorough clinical examination and BIA measurement.

### Inclusion and exclusion criteria

Patients had to be at least 18 years old at inclusion and suffer from signs and symptoms of acute HF with NYHA Class ≥ II, irrespective of LV ejection fraction. The patients were excluded if they had an end-stage renal disease or new need for dialysis, a recent embolic event, complex ventricular arrhythmias at rest, severe pulmonary instability, hemodynamic instability, LV assist devices, an oncologic diagnosis, pregnancy or obesity of class II or higher (body mass index > 35).

### Bioimpedance analysis

BIA measures the “electrical volume” of a body compartment. The impedance of the body is assessed in relation to its length, configuration, cross-sectional area, and the applied signal frequency and then yields raw data in the form of reactance (R), resistance (Xc) and phase angle (PA), as recently reviewed [28]. The volume of the body fluid component is mostly reflected by the resistance, while reactance is rather related to cell membrane integrity. Impedance is expressed as a function of resistance and reactance. The phase angle (PA) is the arc tangent of Xc/R and is related to the ability of cells to function as capacitors, which depends on the integrity of the cell membrane and cellular health [28].

**Fig. 1:**
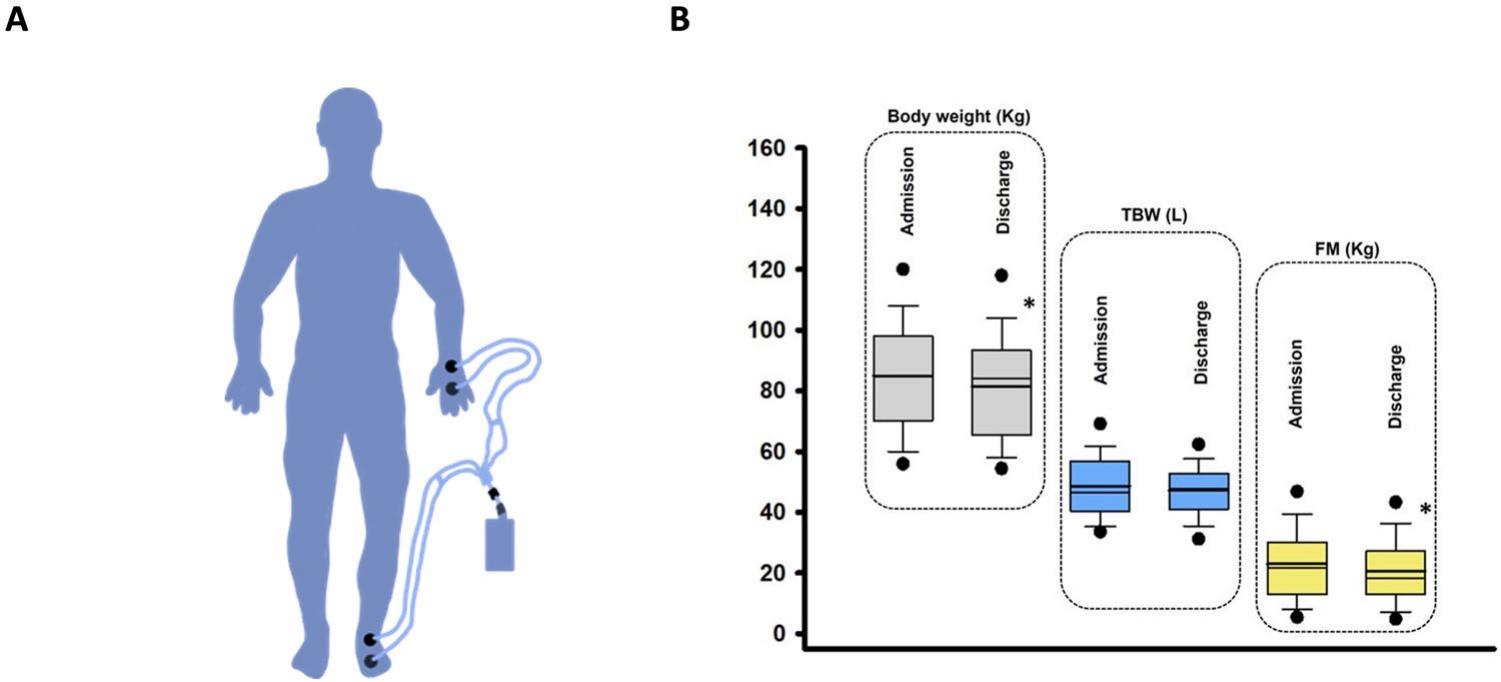
Graphical depiction of the bioimpedance measurement and data on body weight and total body water. **A** Participants were instructed to remain in a supine position with arms and legs separated from the trunk by about 30% to 45% for at least 5 min. Electrodes were attached to the right hand and foot. **B** A significant reduction was observed at discharge as compared to the admission regarding body weight and fat mass but not total body water. The thick line represents the mean, the thin line the median. TBW = total body water; FM = fat mass; *p < 0.05

In our study, whole-body BIA measurements were taken daily using the ©Akern “BIA 101 BIVA” bioimpedance analyzer. The participants were instructed to remain in a supine position with arms and legs separated from trunk by about 30  to 45  for at least 5 min and the skin was cleaned to ensure good contact of the electrodes which were attached to the right hand and foot, in accordance with the standard protocol of the National Institutes of Health technology assessment conference statements [29] Fig. 1A. The device used utilizes a small current of 800 µA at 50 kHz that flows through the body. The acquired data were then processed using ©Akern’s “bodygram.cloud” website. The software uses the standard measured variables in bioimpedance, namely, resistance (R), reactance (Xc), and phase angle (PA) in combination with clinical data such as age, sex, height, and weight. From this set of variables, the software calculates a precise estimate of body composition parameters such as fat mass (FM), fat free mass (FFM), total body water (TBW), extracellular water (ECW), and intracellular water (ICW). These parameters were calculated using the algorithms provided by the manufacturer, which are based on a regression equation that was developed using data from a large population of healthy individuals.

### Plasma and blood volume estimation

We calculated Plasma Volume (PV) as follows [30]:

$$PV=\left(1-hematocrit\right)\times (a+[b\times {BW}_{(Kg)}])$$

where ${a}_{(male)}=1530$ and ${a}_{(female)}=864$; ${b}_{(male)}=41$ and ${b}_{(female)}=47.9$.

Total Blood Volume (TBV) was instead calculated as follows:

$$TBV=\frac{PV}{1-hematocrit}$$

### Transthoracic echocardiography

Resting echocardiographic measurements were measured by a cardiologist in compliance with the American Society of Echocardiography guidelines [31]. For two-dimensional (2D) dimensional and tissue Doppler images, three consecutive beats for patients in sinus rhythm and at least 5 beats for patients with atrial fibrillation were measured and averaged. Left Ventricular (LV) volumes and ejection fraction (EF) were assessed using the biplane method of disks. LV dimensions were measured from frozen end-diastolic and end-systolic images in the parasternal long axis. Relative wall thickness (RWT) was calculated by the formula [32] $$RWT= \frac{(2 \times posterior wall thickness)}{LV diastolic diameter}$$. LV mass was measured by the ASE convention and subsequently indexed to body surface area (LVMI). The early to late transmitral flow velocity ratio (E/A) and E to early diastolic mitral annular velocity (E/e’) were measured by tissue Doppler images.

### Statistical analysis, procedures, and modelling

The analysis of data was performed using the Statistical Package for the Social Sciences Windows version 26.0 (SPSS, Chicago, Illinois, USA). Data are expressed as percentage, mean SD, or median with interquartile range (IQR) for skewed distributions. Normality assumptions were demonstrated with histograms, Q–Q plots, Skewness and Kurtosis, Kolmogorov/Smirnov testing. Descriptive statistics for qualitative categorical variables was performed with frequencies. Data between admission and discharge were compared with t-test for continuous variables and with Chi-square in case of binary ones. All *p* values were considered significant if less than 0.05. The association between parameters as well as between parameters and the composite endpoint was assessed by linear regression analysis. A correlation matrix displayed as a heatmap showing the linear correlation between different variables has been adopted. We utilized stochastic search variable selection (SSVS) to select variables to use in a multiple linear regression model. SSVS is a Bayesian variable selection approach that describes the relative importance of variables (i.e., inclusion probabilities), accounting for uncertainty in other variables that may be used in the model [33]. Through sampling thousands of possible regression models, SSVS determines which variables have the highest probability of inclusion in the models that are best fitting. SSVS then provides marginal inclusion probability scores for each possible variable, which shows the proportion of times that each variable was included in sampled models. Variables with higher inclusion probabilities have more stable associations with the outcome. We then utilized a neural network analysis with two main options, radial base and multilayer perceptron, to unveil the most important parameters able to predict the composite endpoint at 6 months both at the time of admission and at the end of hospitalization (upon being discharged). Factor importance measured by random forest margin of ensemble learning methods was first proposed in 1998 to explain the success of boosting type algorithms [34]. The concept was then generalized to analyze other types of ensemble classifiers such as the random forest model. The classical margin of a sample × in the random forest model is the normalized by 100% based on the predictor of importance. Values less than 80% were not considered important.

## Results

Fifty-one (51) ADHF patients were recruited between June 2020 and October 2021. Two (2) patients were excluded from the analysis due to intrahospital death. Patients (49) were discharged after a median hospitalization time of 7 [4–10] days. At follow-up, six patients died (12%), 4 of which from cardiovascular death (8%). Sixteen patients of the initial cohort (33%) had been hospitalized for HF during the follow-up. In summary, 20 out of 49 patients (40%) had reached the composite endpoint of either cardiovascular death or re-hospitalization for HF at 6 months.

### Baseline characteristics

Baseline characteristics for the study cohort are presented in Table 1. Forty-one patients were male (84%) and were on average 73 ± 11 years old. At admission, patients were mostly affected by HF with reduced EF (HfrEF, 61%) followed by HF with preserved EF (HFpEF, 31%) and HF with mid-range EF (HFmrEF, 8%). Thirty-four (69%) patients had already a diagnosed CHF, while 15 (31%) were diagnosed with de novo HF at admission. A total of 21 (43%) patients presented atrial fibrillation (Afib) at admission. Patients displayed the most common HF comorbidities, i.e. anemia (33%), diabetes (31%), dyslipidemia (60%) and hypertension (98%). A positive family history of cardiac disease was noted in 76% of patients. A total of eight patients had already an ICD (16%), two of which were equipped with a CRT-D system. A CRT-P system was present in two patients (4%).

**Table 1:** Baseline characteristics at admission

Baseline characteristics at admission	
	(*n* = 49)
Age, years	73 ± 11
Men, no. (%)	41 (84)
Body mass index, kg/m^2^	27.3 ± 4.5
Waist circumference, cm	108 ± 15
HFrEF (%)	30 (61)
Comorbidities, no. (%)	
Diabetes	15 (31)
Obese, no. (%)	13 (27)
Hypertension	48 (98)
Atrial Fibrillation	27 (55)
Dyslipidemia	29 (59)
Family history of cardiovascular disease	37 (76)
Medications, no. (%)	
ACEi/ARB	12 (25)
ARNI	12 (25)
β-blocker	42 (86)
Antihypertensive > 1 drug	27 (55)
Diuretics	35 (71)
Antidiabetic	46 (23)
Echocardiography	
LVEF, %	40 ± 16
LV end-diastolic volume index, ml/m2	71 ± 35
LV end-systolic volume index, ml/m2	50 ± 22
RWT	0.44 ± 0.15
LVMI, g/m^2^	128.7 ± 39.3
E/A ratio	2.1 ± 1.4
E/e’ ratio	17 ± 8
TAPSE, mm	18 ± 5
RV s’, cm/s	11 ± 4
VCI, mm	25 ± 4

Data are expressed as mean ± standard deviation or as total and percentages in parentheses

*ACEi* angiotensin-converting enzyme inhibitor, *ARB* angiotensin receptor blocker, *HFrEF* heart failure with reduced ejection Fraction, *LVEF* left ventricular ejection fraction, *LVMI* left ventricular mass index, *RWT* relative wall thickness, *RV s`* right ventricular peak systolic tissue velocity at the tricuspid annulus, *TAPSE* tricuspid annular plane systolic excursion, *VCI* vena cava inferior

The medication at admission is also summarized in Table 1. Beta-blockers had the highest prevalence (84%), followed by ACE-Inhibitors or ARBs (25%), and ARNI (25%). The percentage of patients on diuretic therapy, either loop or thiazides, was 71%. More than half of the patients were on more than one type of antihypertensive drug.

### Cardiac structure and function at admission

The echocardiographic parameters are presented in Table 1. The mean LVEF in the study population was 40 ± 16%, with a LV concentric hypertrophy as shown by a slightly increased RWT combined with an increased LVMI. BSA-indexed LVEDV and LVESV were within the normal to slightly increased range. Right ventricular systolic longitudinal function was preserved as shown by TAPSE and S’ RV. The mitral inflow profile and E/e' ratio were elevated, hinting towards increased LV filling pressures.

### Invasive procedures during hospitalization and at 6 month follow-up

Twenty-four patients received a total of 27 invasive procedures during the study period. 17 interventions were performed during the observed hospitalization and 10 during the follow-up. In total, five patients underwent diagnostic cardiac catheterizations and four patients received a coronary intervention during the hospital stay. One coronary revascularization was carried-out during follow-up. Furthermore, three CRT-Ps and three ICDs were implanted during the initial stay while two CRT-Ds were implanted during follow up. One patient received an ablation due to Afib during the initial hospitalization, while three patients received it during the follow-up period. One tricuspid valve clip was implanted in two patients, one during the initial stay and the other during follow-up. Lastly, one biological aortic valve implanted via TAVI, one mitral valve clip, and one LAA-Occluder were implanted during follow-up.

### Clinical course

The differences observed between admission and discharge of the most relevant clinical, HF-related, and laboratory data are summarized in Table 2. As compared to the admission, patients at discharge displayed lower BW Fig. 1B, lower NT-proBNP, lower systolic and diastolic blood pressure (SBP and DBP, *p* < 0.0001 and *p* = 0.003, respectively), as well as lower heart rate (HR, *p* = 0.017). At hospital discharge patients displayed improved NYHA class, a lower prevalence of peripheral edema and pulmonary congestion. There were no differences in electrolytes, renal function and blood count between admission and discharge. As compared to the admission, both PV and TBV decreased significantly at discharge (*p* = 0.045 and *p* < 0.0001, respectively).

**Table 2:** Comparison of patients’ characteristics at hospital admission versus hospital discharge

*n* = 49	Admission	Discharge	*p*-value
Physical features			
Body Weight, Kg	85 ± 19	82 ± 18	** < 0.0001**
Body mass index, Kg/m^2^	27 ± 5	26 ± 4	** < 0.0001**
Heart rate, beats/min	76 ± 15	71 ± 12	**0.017**
Systolic blood pressure, mmHg	124 ± 22	115 ± 19	** < 0.0001**
Diastolic blood pressure, mmHg	75 ± 11	68 ± 11	**0.003**
Heart failure features			
NYHA Class	3 ± 0.4	2 ± 0.5	** < 0.0001**
Peripheral edema, %	36 (74)	9 (18)	** < 0.0001**
Pulmonary congestion, %	10 (20)	0 (0)	** < 0.0001**
Laboratories			
NT-proBNP, pg/mL	4469 [2416–7791]	2985 [1407–5934]	**0.003**
Sodium, mmol/L	139 ± 4	139 ± 3	0.881
Potassium, mmol/L	4.2 ± 1	4.1 ± 1	0.837
Creatinine, mg/dL	1.5 ± 0.7	1.4 ± 0.8	0.264
eGFR, mL/min/1.73 m^2^	52 ± 23	55 ± 22	0.095
Hemoglobin, g/dL	13 ± 2	12 ± 2	0.425
Hematocrit, %	0.38 ± 0.1	0.37 ± 0.1	0.490
Plasma volume, mL	3126 [2646–4142]	3005 [2666–4254]	**0.045**
Blood volume, mL	5015 [4408–7065]	4954 [4195–6819]	** < 0.0001**

NYHA Class, New York Heart Association Class; eGFR, estimated glomerular filtration rate; Data are expressed as mean ± standard deviation, as total and percentages in parentheses or as median and interquartile ranges. A p-value < 0.05 was considered significant

### Bioimpedance measurements

Table 3 summarizes the bioimpedance measurements at admission and discharge. Measured parameters, such as resistance, reactance, and phase angle, did not change significantly over the course of hospitalization Table 3 and Supplemental Fig. 1. As compared to the admission, FM and FFM were significantly lower at discharge, while TBW was not (*p* = 0.083, Table 3 and Fig. 1B). Other BIA-derived parameters displayed no change over the time course of hospitalization. No adverse events due to the measurements performed with BIA were observed, in particular patients carrying ICDs or Pacemakers were unaffected by the measurement.

**Table 3:** Bioimpedance parameters at hospital admission versus hospital discharge

*n* = 49	Admission	Discharge	*p*-value
Measured parameters			
Resistance, Ohm	457 ± 103	465 ± 89	0.456
Reactance, Ohm	41 ± 16	42 ± 15	0.932
Phase Angle, °	5 ± 2	5 ± 2	0.920
Derived parameters			
Fat mass, Kg	23.0 ± 13	20.5 ± 11	**0.008**
Fat mass %	26 ± 10	24 ± 10	**0.045**
Fat free mass, Kg	62.0 ± 12	61.0 ± 12	0.706
Fat free mass %	74 ± 10	76 ± 10	**0.045**
Total body water, L	48.6 ± 11	47.2 ± 9	0.083
Total body water %	58 ± 9	59 ± 8	0.121
Extracellular water, L	25.1 ± 6	23.8 ± 5	0.130
Extracellular water %	51 ± 8	51 ± 9	0.549
Intracellular water, L	23.5 ± 7	23.4 ± 7	0.797
Intracellular water %	49 ± 9	49 ± 9	0.866
ECW/ICW	1.10 ± 0.3	1.24 ± 0.7	0.184
ECW/TBW	0.51 ± 0.1	0.69 ± 0.3	** < 0.001**

Data are expressed as mean ± standard deviation. A *p* value < 0.05 was considered significant

*ECW* extracellular water, *ICW* intracellular water

**Fig. 2:**
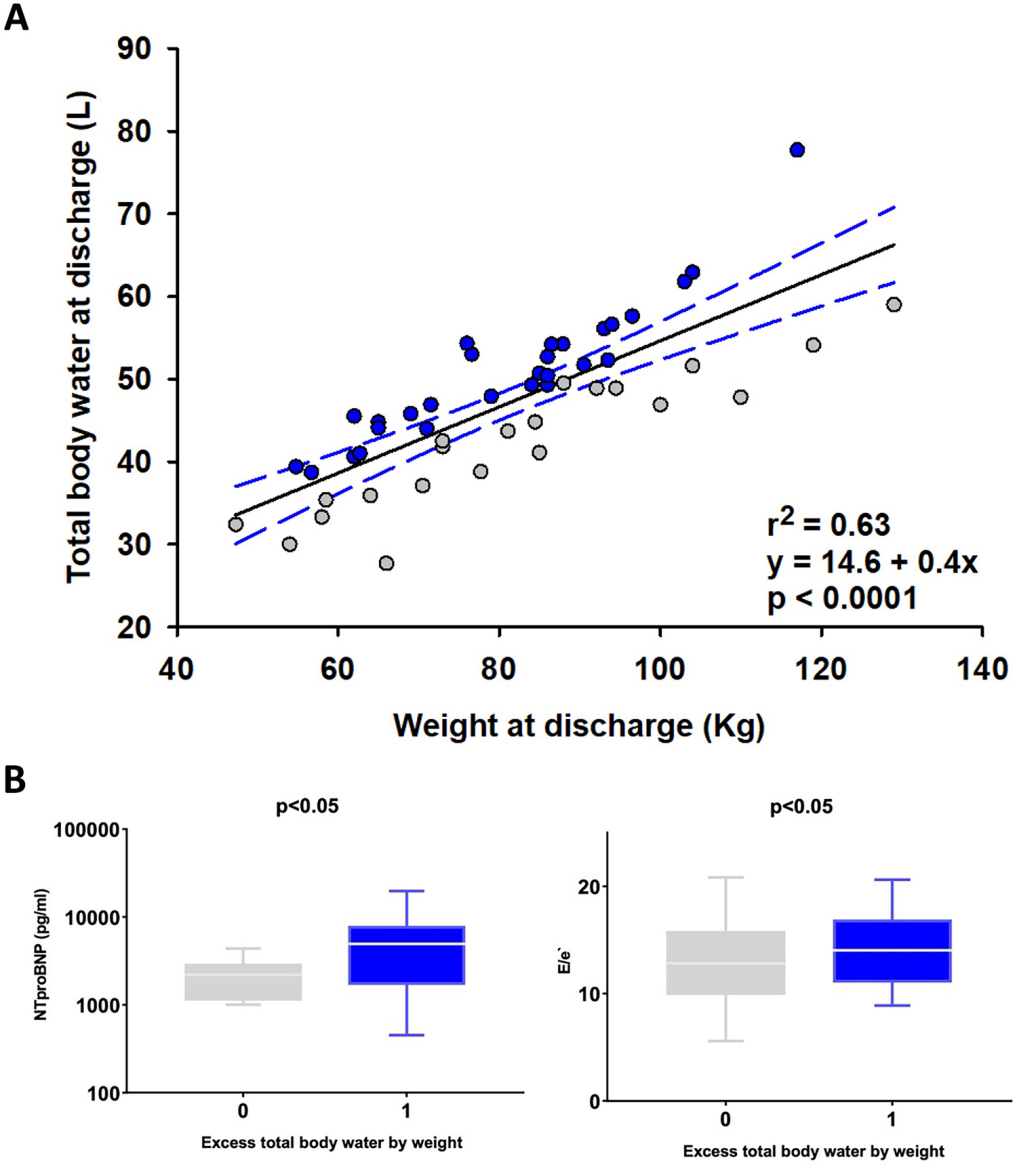
Relationship between total body water and body weight at discharge and the excess total body water as predicted by weight. **A** Linear regression analysis investigating the association between total body water (TBW) and body weight (BW) at discharge. Patients with TBW less than or equal to that predicted by BW are represented in grey (*n* = 21), those with TBW higher than expected by weight are represented in blue (*n* = 28). As compared to patients with TBW less than or equal to that predicted by BW, those with TBW levels in excess of that predicted by BW had higher NT-proBNP as well as E/e`

TBW at discharge was directly correlated with BW (Fig. 2A, *r*^*2*^ = 0.63, *p* < 0.0001). To further explore relationships between TBW independent of BW, we next compared patients according to expected TBW values based upon BW (using the least squares linear regression equation displayed in Fig. 2A). At discharge, patients with TBW levels exceeding those predicted by BW showed significantly higher values of NT-proBNP and E/e´ (Fig. 2B, blue boxes) compared to subjects whose TBW was less than or equal to the expected ones (Fig. 2B, grey boxes). In line with this, patients with excess TBW at discharge showed a higher prevalence of NYHA class III (Supplemental Table 1) compared to patients with TBW values below or equal to the expected values by BW.

### Heatmap correlation graph

**Fig. 3:**
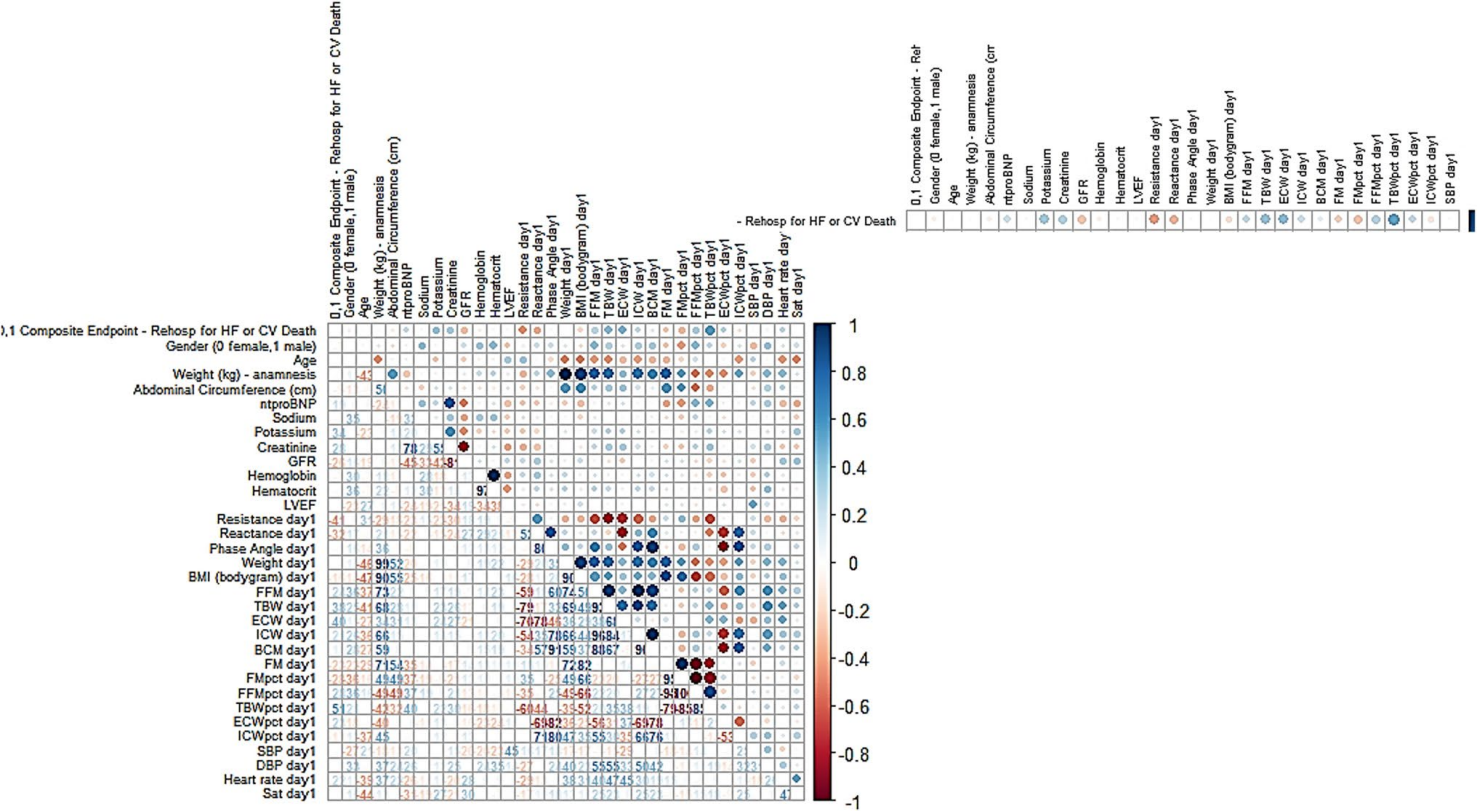
Heatmap correlation between parameters at admission and the composite endpoint. A moderate to high correlation is observed between TBW and ECW and the composite endpoint. The colored column on the right side displays the R^2^ ranging from -1 (red) to 1 (blue). BMI = body mass index; DBP = diastolic blood pressure; ECW = extracellular water; FM = fat mass**;** FFM = fat free mass; GFR = glomerular filtration rate; ICW = intracellular water; LVEF = left ventricular ejection fraction; TBW = total body water; SBP = systolic blood pressure

We then performed a circle heatmap analysis to show the correlations between different parameters and between parameters at admission and occurrence of the composite endpoint (Fig. 3). Based on the analysis, the highest correlation was achieved by TBW% (*r* = 0.459, *p* < 0.05). Creatinine and potassium also turn out to be correlated, while GFR, resistance, reactance, and FM% were inversely correlated. The heatmap then also shows correlations between other included parameters of the predictor pattern, showing an expected high correlation among BIA parameters. Interestingly, body weight showed a poor correlation for the composite endpoint (*r* = 0.026, *p* = 0.883).

### Multivariate survival analysis

With the aid of machine learning tools, several Cox hazards multivariate models were derived in this study using the potential factors with measurements recorded at admission and the associated delta changes during the hospitalization period. Because delta total body water (ΔTBW) and delta Resistance (ΔResistance) are highly correlated (*r* = − 0.94, *p*-value < 0.001), the two variables were considered disjointly for building the prediction models. Five of the developed models are displayed in the Supplemental Table 2. These models showed statistical significance of *p* < 0.05 and reasonably satisfied all assumptions of Cox hazards regression. Predictors with individual statistical significance within all the derived models (wherever included) were ΔTBW, ΔResistance, ΔReactance, and ΔSaturation. Furthermore, ΔFM has revealed significant association with the outcome at level of significance 0.1 or less in all models where ΔTBW was considered a potential predictor. Delta TBW and delta resistance were highly correlated (*r* = − 0.94, p-value < 0.001) and revealed the most significant associations with the outcome when considered disjointly in the developed models. The only predictor with individual statistical significance within all the derived models was delta Reactance.

Based on AIC goodness-of-fit estimator, the “best” Cox Hazards prediction model was Model 3, which included four significant variables, namely: ΔTBW, ΔFM, ΔReactance, and ΔSaturation, beside some other potential predictors: delta hemoglobin (ΔHb), delta potassium (ΔPot), and nt-proBNP ≥ 1000 measured at admission. The associated regression coefficients, hazard ratios and the corresponding 95% confidence intervals are displayed in Table 4. In this model, the most significant risk predictor is ΔTBW indicating a very strong relation between total body water change and risk of cardiovascular death and/or re-hospitalizations for HF. A one unit increase in ΔTBW decreased the hazard ratio by a factor of about 23%. In addition, a one unit increase in ΔFM decreased the hazard ratio by a factor of 15%. The hazard ratio was decreased by 9% and 32% for patients with a unit increase in ΔReactance and ΔSaturation, respectively. Finally, patients with an Nt-proBNP ≥ 1000 were about 3.3 times as likely to reach the composite endpoint. Delta body weight was removed from the analysis to avoid collinearity problems as it was found to have a moderate correlation with DTBW (rho = 0.6), a poor one with delta resistance (rho = − 0.48) and was not found significant in most of the models.

**Table 4:** Estimates from the “best” multivariate Cox hazards model (Model 3) independently associated with the composite endpoint at 6 months

Variables	β (SE)	HR (95% CI)	*p*-value
Bioimpedance variables			
Δ total body water	− 0.254 (0.07)	0.776 (0.67, 0.89)	**0.0006**
Δ Reactance	− 0.096 (0.04)	0.909 (0.84, 0.98)	**0.016**
Δ fat mass	− 0.163 (0.08)	0.849 (0.72, 0.99)	**0.048**
Clinical variables			
Δ hemoglobin	− 0.308 (0.20)	0.735 (0.49, 1.09)	0.131
Δ potassium	− 0.023 (0.24)	0.978 (0.61, 1.56)	0.924
Δ arterial O_2_ saturation	− 0.378 (0.15)	0.685 (0.52, 0.91)	**0.009**
nt-proBNP ≥ 1000	− 1.195 (1.12)	0.302 (0.03, 2.73)	0.287

The Δ variables represent the absolute difference observed between discharge and admission; Nt-proBNP ≥ 1000 ng/L measured at admission. The model Akaike Information Criterion (AIC) goodness-of-fit estimator = 101.4

*SE* standard error, *HR* hazard ratio, *CI* confidence interval

### Risk prediction model

To further characterize the role of BIA-derived TBW in the context of ADHF, we investigated the most relevant clinical variables able to predict the occurrence of major cardiovascular events at 6 months in the current cohort of patients. To increase accuracy and exclude potential biases, we decided to identify the population at higher risk of experiencing the composite endpoint at specific timepoints, while considering the effect of the hospitalization on the clinical course. Since NT-proBNP is an established prognostic risk factor for HF patients, we selected the subgroup of patients that in our cohort were presenting a NT-proBNP ≥ 1000 pg/mL at admission (*n* = 35).

**Fig. 4:**
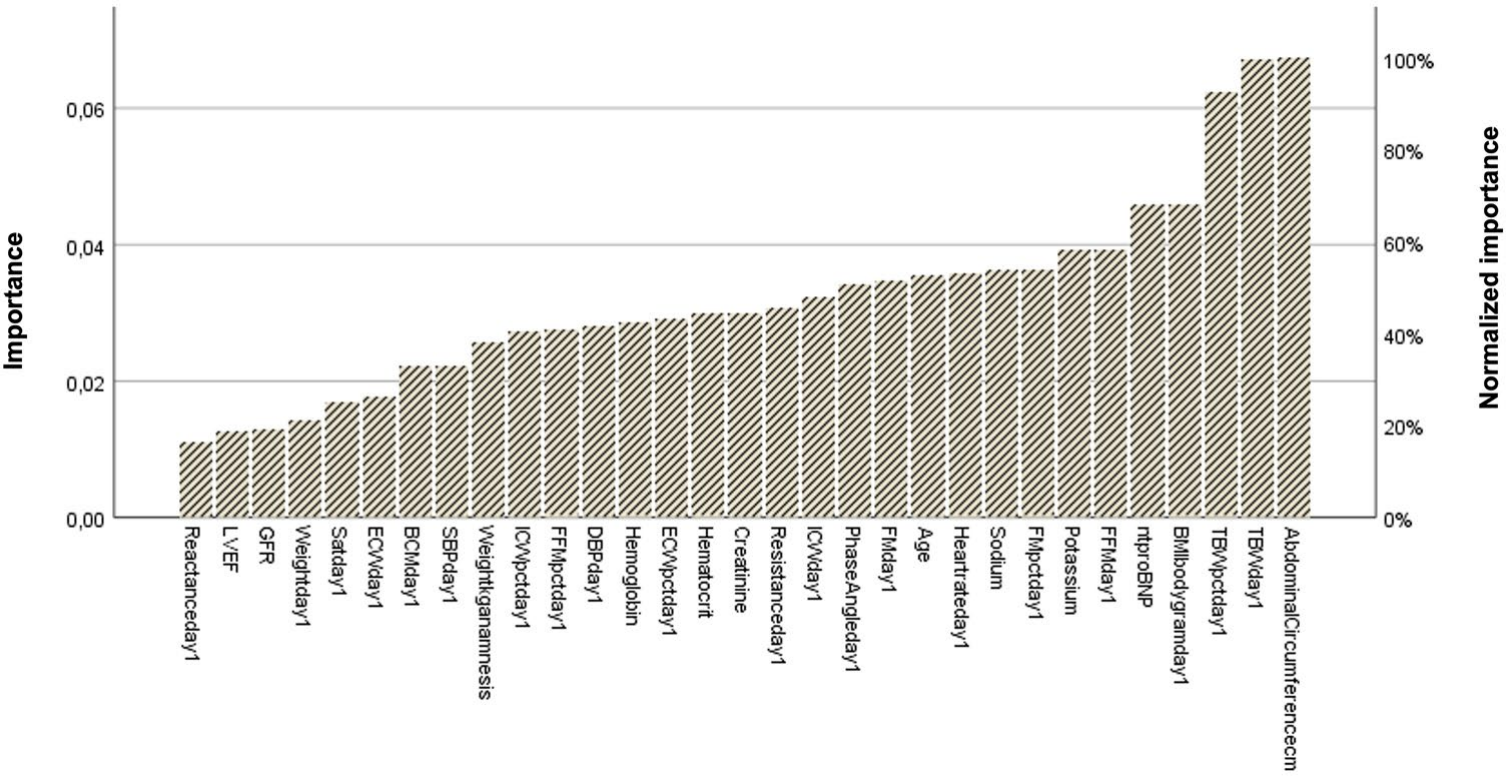
Normalized importance of risk predictors of the composite endpoint observed at admission. From right to left are represented the most important normalized parameters observed at day 1 of hospitalization predicting the composite endpoint at 6 months through neural network analysis

We then utilized a neural network analysis with two main options, radial base, and multilayer perceptron, to unveil the most important parameters able to predict the composite endpoint at 6 months both at the time of admission and at the end of the hospitalization before being discharged. The results are summarized in Fig. 4. At admission, the most relevant parameters that we observed were abdominal circumference and TBW expressed in liters with an importance of 100%, followed by TBW expressed in percentage with normalized importance of more than 80%. BMI and nt-proBNP at admission followed with more than 60% normalized importance each.

## Discussion

In this pilot study, we investigated the relationship between BIA derived TBW and cardiovascular outcomes in ADHF patients followed up over 6 months after hospital discharge. We showed that, as compared to patients with TBW at discharge less than or equal to that predicted by BW, those with TBW levels in excess than predicted had greater evidence of cardiac congestion, i.e. worse NYHA class, higher NT-proBNP as well as E/e´. In addition, reduced ΔTBW between admission and discharge was associated with increased risk for the composite endpoint of HF hospitalization or death over 6 months of follow-up. In line with this, TBW at admission was found to have the highest prediction importance of the combined endpoint for a subgroup of high-risk HF patients in a neural network analysis. Importantly, overhydration in these patients could not be identified based upon clinical assessment alone, emphasizing the role of BIA assessment in this cohort of patients. These data underline the potential of TBW for clinical assessment and prognostic stratification of ADHF patients.

### Bioimpedance assessment and HF

These results are in line and extend previous findings on BIA in HF patients [22, 24–26, 35–37]. Di Somma et al. previously showed that overhydration assessment by BIA in a similar cohort of ADHF patients significantly correlated with the occurrence of death or re-hospitalization at 3 months after hospital discharge [24]. In another pilot study by Valle et al., BIA and BNP guided management of the diuretic treatment allowed to identify patients at higher risk and improved their outcome at 6 months [25]. In the current study, we also demonstrated that a more pronounced reduction in TBW during hospitalization was associated with a lower occurrence of major cardiovascular events. This is in line with a previous study, which reported [38] a clinically prognostic additive value of BIA measured resistance at 90 days [38]. Interestingly, in our study BW was once again not able to properly reflect the volume status of ADHF patients, as previously described [15, 23]. This could be explained by a significant decrease of FM and FFM during the hospital stay, as important contributors of BW decrease. We interpret the presented data as a result of several factors contributing to relative rapid change in body composition in these patients. One of them is the severe catabolic state: acute illness, including heart failure exacerbations, can induce a catabolic state where the body breaks down tissues, including fat and muscle, at an accelerated rate [39–41]. Moreover, a sarcopenic process during an episode of ADHF has been already described in the literature and is mostly due to the inactivity, reduced appetite leading to a caloric deficit stress and change in diet leading up to poor nutrition in an acute setting [39–41]. Nonetheless, the data on the assessment of FM and FFM measurements need to be interpreted with caution since those can be affected by hydration status, which fluctuates significantly in HF patients.

Eventually, a lack of correlation between BW loss and fluid balance is possibly due to numerous in-hospital confounders, including the lack of a reliable system for documentation of diuresis [42]. This concept underlines once again the limitations of BW monitoring for fluid management during the treatment of hospitalized ADHF [42].

### Risk prediction model

Of the established HF risk prediction scoring models, the Seattle Heart Failure Model (SHFM) [43], and the META-Analysis Global Group in Chronic Heart Failure (MAGGIC) risk model [44] have shown the highest validity. However, both models fall too short in supporting clinical management in ADHF patients, rendering their applications in an acute clinical setting limited [45]. Among high-risk HF patients, prediction models for major cardiovascular events in the phase between 3 and 6 months after hospital discharge are currently missing [45]. Moreover, none of the current risk prediction models focuses on the volume status of the HF patients. The aim of the current investigation was to evaluate a combination of body composition and volume status parameters for prognostic stratification of ADHF patients. The use of clinical and biomarkers data together with BIA assessment has already been shown to provide good results in detecting the presence of fluid overload as well as the risk of developing cardiovascular events. Nevertheless, a risk prediction model has not been clinically implemented yet [38]. The current study extends the evidence of the potential role of BIA derived TBW within a clinical risk model for prognostic stratification of ADHF patients. Further studies in larger cohort of patients could validate a risk prediction score including BIA derived parameters of body composition and its impact on clinical management of ADHF patients.

### Clinical implications

While in an intensive care unit setting volume status changes of ADHF patients is assessed through exact counting of the fluids’ input and output, this method is inconsistently utilized in the peripheral ward. Moreover, fluid balance assessment is not exempted from flaws, i.e. the counting of perspiration which is often a forgotten output parameter. When the patients are discharged at home, they are usually advised to monitor their changes in body weight, considering that an increase of more than 3 kg within a week is generally interpreted as an acute worsening that needs medical attention [46]. However, signs and symptoms may precede a decompensation event while changes in body composition occur before changes in body weight [23, 47]. In HF patients, BIA has been shown definitively helpful in guiding diuretic therapy during a hospitalization and from a clinical perspective this can lead to a shorter and more effective hospital stays [24, 47, 48].

Although many non-invasive monitoring solutions have been studied or are currently under research [49, 50], effective solutions for the assessment of the patient congestion status in home settings are still not available [51]. In homecare settings, BIA has shown promising results [22, 23, 52]. In the study by Cuba Gyllenstein et al., daily measurements of transthoracic BIA using a vest with textile electrodes provided a more accurate indication of upcoming decompensations when compared to body weight [23]. Darling et al. was able to show that a wearable vest employing BIA was highly sensitive and specific for identifying recurrent HF events [22]. However, conclusive data from large clinical trials on the role of BIA in home care settings for the prevention of re-hospitalizations and mortality are still lacking. Bioimpedance analysis alone may not be enough and a more comprehensive assessment of the patients is needed in home care settings [13], whether this regards a multiparameter monitoring [36] or a more complex algorithm considering various characteristics of the patients [53]. From a clinical perspective, the daily assessment of the patient health status comprising a dedicated clinical questionnaire and assessment of different parameters including body composition through home monitoring may be pivotal for the prevention of acute exacerbations of the chronic congestion.

## Limitations

The data presented in this study were collected from a single medical center, therefore potentially leading to a selection bias. The small sample size is another limitation of the study and larger clinical studies are needed to confirm the current findings.

The accuracy and precision of BIA measurements may vary significantly based on several factors such as variations in limb length (usually estimated from body height), recent physical activity, nutrition status, tissue temperature and hydration, blood chemistry, ovulation and electrode placement and this represent a known intrinsic limitation of the technique [17, 18]. Patients were not required to fast or to have the same level of mobilization before the measurements. Being an observational study and due to the concomitant clinical routine, the measurements were often taken at different times of the day and independently of the medications administered. All those aspects may have influenced the measured BIA parameters.

## Conclusions

In ADHF patients, BIA-derived TBW is associated with cardiovascular adverse events over 6 months, with TBW at admission having the highest prediction importance in a subgroup of high-risk HF patients. Overhydration in these patients at discharge could not be identified based upon clinical assessment alone, emphasizing the importance of BIA assessment in the intrahospital setting. The role of BIA for clinical management and prognostic stratification of HF patients merits further investigation.

## Data Availability

The data that support the findings of this study are not openly available due to reasons of sensitivity and are available from the corresponding author upon reasonable request.

## Abbreviations

AIC: Akaike information criterion
BIA: Bio-impedance analysis
ECW: Extracellular water
FM: Fat mass
FFM: Fat-free mass
ICW: Intracellullar water
PA: Phase angle
SSVS: Stochastic search variable selection
